# Engagement of vulnerable youths using internet platforms

**DOI:** 10.1371/journal.pone.0189023

**Published:** 2017-12-20

**Authors:** Melissa Chan, Tim M. H. Li, Yik Wa Law, Paul W. C. Wong, Michael Chau, Cecilia Cheng, King Wa Fu, John Bacon-Shone, Qijin Emily Cheng, Paul S. F. Yip

**Affiliations:** 1 Department of Social Work and Social Administration, The University of Hong Kong, Pokfulam, Hong Kong; 2 Department of Rehabilitation Sciences, The Hong Kong Polytechnic University, Hung Hom, Hong Kong; 3 The Hong Kong Jockey Club Centre for Suicide Research and Prevention, The University of Hong Kong, Pokfulam, Hong Kong; 4 School of Business, Faculty of Business and Economics, The University of Hong Kong, Pokfulam, Hong Kong; 5 Department of Psychology, The University of Hong Kong, Pokfulam, Hong Kong; 6 Journalism and Media Studies Centre, The University of Hong Kong, Pokfulam, Hong Kong; 7 Social Sciences Research Centre, The University of Hong Kong, Pokfulam, Hong Kong; Maastricht University, NETHERLANDS

## Abstract

**Aim:**

The aim of this study was to explore the online distress and help-seeking behavior of youths in Hong Kong.

**Methods:**

A cross-sectional telephone-based survey was conducted among 1,010 young people in Hong Kong. Logistic regression analysis was then performed to identify the factors associated with those who reported expressing emotional distress online and the differences in help-seeking behavior among four groups of youths: (1) the non-distressed (reference) group; (2) “Did not seek help” group; (3) “Seek informal help” group; and (4) “Seek formal help” group.

**Results:**

The seeking of help and expression of distress online were found to be associated with a higher lifetime prevalence of suicidal ideation. The “Seek formal help” and “Did not seek help” groups had a similar risk profile, including a higher prevalence of suicidal ideation, non-suicidal self-injury, unsafe sex, and being bullied. The “Seek informal help” group was more likely to express distress online, which indicates that this population of youths may be accessible to professional identification. Approximately 20% of the distressed youths surveyed had not sought help despite expressing their distress online.

**Implication:**

The study’s results indicate that helping professionals have opportunities to develop strategic engagement methods that make use of social media to help distressed youths.

## Introduction

Mental health of young people demands the attention of both practitioners and policymakers, as adolescence is the period in which many mental health disorders are first detected. According to a review paper [1], research studies around the world show a prevalence of mental health disorders ranges from 8% to 27% among young people aged 24 and below. To prevent or mitigate the adverse impacts of mental health problems, service providers need to find ways to connect youths with mental health needs to mental health services to facilitate early identification and intervention. However, the range of barriers to mental health service access by this population [1] highlights the significant gap that exists between service needs and service utilization.

To reduce that gap, it is crucial that practitioners understand the influences on barriers to service use [2]. The various models used to explain patterns of service utilization adopt different perspectives to explain those barriers. For example, the behavioral model, which views healthcare decisions as a rational process [3], emphasizes the physical barriers to mental health service access, such as demographic factors (e.g., receiving a low level of education or being a member of an ethnic minority) and a lack of enabling resources (e.g., a lack of service information and inaccessible service locations) [4]. Another widely used model, the Health Belief Model (HBM), suggests that healthcare decisions are based on self-perception rather than an objective medical condition [5]. Therefore, the barriers to service utilization stem from subjective experiences and health beliefs. They perceived such barriers include a fear of treatment, lack of confidentiality, and the stigma associated with mental illness [6]. Different from the behavioral model’s assumption that individuals are rational decision-makers, some models assume that healthcare decisions can be influenced by peers and family members. According to the Network Episode Model (NEM) [7], for instance, service barriers are more likely to be influenced by social factors than individual factors. In addition, the Gateway Provider Model (GPM) extends the NEM to describe the role of the “gateway provider” in influencing service use by young people. Gateway providers direct youths toward and connect them with services, and the GPM thus highlights the important influence that helping professionals exert over service utilization [8].

Internet platforms may serve as gateway providers. A 2017 report by the International Telecommunication Union stated that 70% of the world’s youth are online. In developed countries, even 94% of young people aged 15–24 use the Internet [9]. In Hong Kong, where the current study was conducted, the household Internet penetration rate in that year was 82.8% [10]. The high global and local Internet penetration rates suggest that the provision of mental health services over the Internet may be a viable option, as it would reduce the physical barriers to service access via enhanced availability, accessibility, and affordability [11]. In addition, the Internet could offer a secure service channel, thereby reducing the psychological barriers to service access (e.g., stigma associated with mental illness) perceived by many young people [12].

According to both the behavioral model and HBM, Internet services can bridge the gap between youths’ mental health needs and service access because they reduce the physical and psychological barriers to such access. However, a danger is that the social influences that prevail in the cyber world may encourage them to seek inappropriate solutions to their problems. For example, young people engaged in sex work have formed strong collaborative networks online to share precautionary tips on sex-related problems through social media rather than seeking professional help [13]. Examples abound of social media serving as platforms for the spread of problem behaviors. For instance, online suicide groups on social media that provide detailed descriptions of suicide methods may encourage suicidal thoughts and even promote suicidal acts [14]. However, social media could also serve as a platform for helping professionals to develop proactive engagement and health promotion strategies and encourage youths to seek professional help [15].

To date, helping professionals’ involvement in social media-based mental health interventions has been minimal, which researchers have attributed to the low adherence rate to such interventions [16]. Although there is a long history of online counseling services, those services have not reached out to vulnerable young people [17]. To the best of our knowledge, there is only one evaluation study on Internet outreach services providing HIV screening for LGBT youths [18], and yet their service needs remain undetermined. The GPM suggests that online helping professionals can be proactive gateway providers that identify young people experiencing problems and recommend them to services [19]. The study reported herein was to investigate the possibility of reaching youths who need help via various Internet platforms (e.g., social media, Internet forums).

The study’s specific aims were to (1) better understand the behavior of young people disclosing their distress on the Internet; (2) elucidate the characteristics of youths who make such online disclosures to allow helping professionals to identify and engage with them; and (3) examine the differences between those who are at risk but do not seek help and those who do seek help. Its results will help professionals to devise a more strategic outreach approach [20]. The study was a response to the call for research examining whether Internet platforms such as social media can serve as a substitute for traditional face-to-face psychosocial services and connect at-risk youths with such services.

## Method

### Data collection

A cross-sectional telephone survey was conducted in Hong Kong between January 23 and March 22, 2013. A large sample of 80,000 mobile phone numbers was randomly generated using the mobile number prefix data published by the Hong Kong Office of the Telecommunications Authority. Among the 80,000 numbers, 8,912 were not in our desired age group. The response rate was 35.4%, giving us a total of 1,010 respondents. Details of the research methodology and study measurements can be found in [21]. Prior ethical approval was obtained from the Human Research Ethics Committee for Nonclinical Faculties at the University of Hong Kong.

### Measures

#### Dependent variables

Two dependent variables were used in our analysis. First, the respondents were asked whether they had ever made an online disclosure of distress on online platforms such as blogs, forums, or microblogs (e.g., Twitter or Weibo [a popular Chinese microblogging site]) and social networking sites (e.g., Facebook). This dependent variable (DV1) is a categorical variable (yes vs. no).

Second, for the second dependent variable (DV2) the respondents were classified into four groups, namely, a non-distressed group and three distressed groups. Their level of distress was self-evaluated on the basis of seven life situations occurring over the month prior to the survey. These situations pertained to their academic situation, job, finances, social networks, health, family relationships, and relationships with their spouse or partner.

For those in the distressed groups, they were classified as the “Did not seek help” group, “Seek informal help” group, and “Seek formal help” group, depending on their reported help-seeking behavior. Informal help included help from family, relatives, friends, classmates or colleagues, and teachers, whereas formal help included help from social workers, counselors, clinical psychologists, psychiatrists, general practitioners, or paraprofessionals (e.g., psychiatric nurses, occupational therapists).

**Fig 1:**
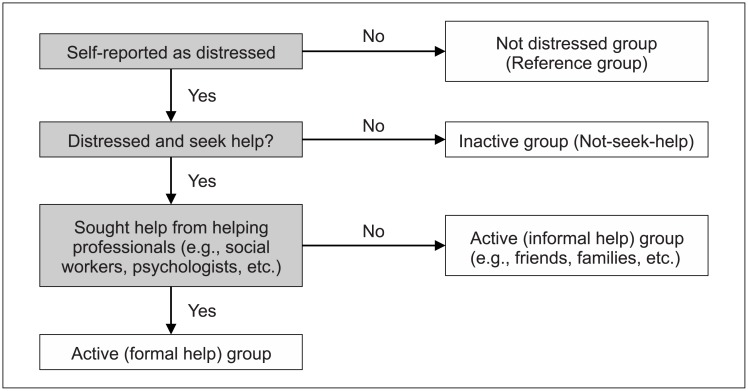
Categorization of the four groups for DV2.

DV2 is also a categorical dependent variable (did not seek help vs. seek informal help vs. seek formal help). If a respondent had sought both formal and informal help, he or she was categorized as a member of the “seek formal help” group. (Please refer to Fig1.tif for Fig 1. Categorization of the four groups for DV2.)

#### Independent variables

The study’s independent variables were classified into four domains. The first was sociodemographic characteristics, including sex (male or female), age (below 18, 18 to 24, or 25 to 29), education level (Secondary 6 or above, which is equivalent to U.S. Grade 12 or above), and student status (student or not).

The second were the psychological factors of health-related quality of life and self-perceived distress (as described in DV2). Health-related quality of life was measured using the 12-item General Health Questionnaire (GHQ-12), which is designed to detect emotional distress in the general population. The reliability of the GHQ-12 is 0.83, and it has demonstrated internal validity with a sensitivity of 93.5% and specificity of 78.5% among general practice patients [22]. The Chinese version used in this study has also been validated [23].

The third set of independent variables were behavioral factors, including Internet usage (hours per week) and lifetime risk behaviors. Risk behaviors were measured using a checklist derived from the Youth Risk Behavior Questionnaire (YRBQ) and a review of the literature on definitions of the term “at risk.” Checklist items included suicidal thoughts, self-harm (without suicidal thoughts), risky alcohol intake, at-risk smoking, substance abuse, financial issues, risky sexual behavior, bullying, and criminal offenses. The YRBS has been validated and used in a local setting [24].

The fourth and final set were social communication and relationship variables, including means of communicating with others, ways of disclosing distress, and interpersonal relationships. The communication methods investigated included online platforms such as blogs, forums, microblogs, and social networking sites and private communication means such as face-to-face conversations, telephone conversations, text messages sent via mobile phone or WhatsApp, and instant messages. The frequency with which each method was used to contact others and disclose distress was measured using five-point (0 = never, 1 = once a week, 2 = several times a week, 3 = once a day, and 4 = several times a day) and four-point (1 = never, 2 = rarely, 3 = sometimes, and 4 = very often) Likert scales, respectively. Furthermore, respondents’ willingness to be approached by helping professionals via online means and their attitudes toward seeking support via the Internet were also evaluated using a five-point Likert scale (1 = least likely; 5 = most likely). The size of a respondent’s social network was assessed by the number of friends in his or her Facebook network.

### Statistical analysis

The study results underwent three stages of statistical analysis. First, descriptive analysis was performed to create a respondent profile. Second, univariate binary logistic regression analyses were conducted with DV1 and adjusted for age and gender. The non-distressed group served as the reference group in these analyses. Sociodemographic characteristics and the other independent variables were analyzed to obtain adjusted odds ratios (OR) and their 95% confidence intervals (CI). Third, univariate multinomial logistic regression analyses were conducted with DV2 and adjusted for age and gender, with the non-distressed group again serving as the reference group and sociodemographic characteristics and the other independent variables analyzed to obtain adjusted OR and their 95% CI. All three stages of analysis were conducted using SPSS software (IBM SPSS Statistics 20, IBM Corporation).

## Results

### Internet usage

The largest proportion of youths surveyed in this study were aged between 18 and 24 (48%). About 27% were younger than 18, and the remaining 26% were between the age of 25 to 29. A large proportion of the respondents were students (58%), and the remainder were employed. Just over half were women/girls (54%).

The respondents reported spending an average of 23.4 hours per week on the Internet (interquartile range: 10 to 30 hours per week). A small proportion (5%) said they spent over 60 hours per week on the Internet. Only 4% of the respondents indicated that they do not use any of four online social platforms (i.e., Facebook, Twitter, online forums such as HK Golden, and blogs such as WordPress), whereas 71% said they use these platforms at least once a day. Four percent stated that they do not display any personal information online, with the majority reporting that they display some such information, such as their name, contact e-mail address, age, marital status, hobbies, and the social networks of friends and family, as well as photos.

Online social platforms not only facilitate communication but also provide a way to express distress. A large majority of the respondents expressed distress via traditional private means of expression such as face-to-face conversations (91%) and phone calls (86%). They also find the internet an open platform for expression of distress. A majority of the respondents (68%) stated that they have disclosed distress on a social networking platform. Yet, a quarter of respondents stated that they do not express distress on online platforms.

With regard to gaining support via the Internet, over 60% of respondents indicated that they talk about their problems with their friends over the Internet, and 62% reported having friends on the Internet with whom they can share their joys and sorrows. A quarter of the respondents indicated that they would be willing to be approached by online professionals.

As noted above, the use of Internet platforms to voice distress was just as common among the youths surveyed as the use of private means of communication. Table 1 below presents the demographics and Internet usage patterns of the respondents. Table 2 presents data on their expression of distress via online platforms and attitudes toward gaining support via the Internet.

**Table 1:** Demographics and usage of Internet platforms of all youths surveyed.

	All participants (N = 1010)		
***Gender***			
Male	46%		
Female	54%		
***Age*** @	Mean 20.79, Median 21		
< 17	27%		
18–24	48%		
25–29	26%		
**Education** @			
Higher education (Grade 12 or above)	54%		
Below Grade 12	46%		
**Employment status**			
Student	58%		
Non-student	42%		
**Internet usage**	Mean 23.4, Median 20		
No internet usage	0%		
Below 10 hours	27%		
10 to 20 hours	26%		
20 to 30 hours	21%		
30 to 40 hours	10%		
40 to 60 hours	10%		
60 hours or above	5%		
**Facebook friends**			
Below 150 friends	30%		
150 to 300 friends	32%		
300 or more friends	38%		
**WhatsApp friends**			
Below 25 friends	26%		
26 to 50 friends	30%		
50 friends or more	44%		
**Usage of online social platforms**			
None at all	4%		
Some usage	96%		
**Display of personal information on online social platforms**			
None at all	4%		
Some information	96%		
**Use of online platforms**	**Use at least once a day**	**Use at least once a week**	**Never use**
- social networking platforms	71%	22%	7%
- microblogging @	34%	17%	48%
- forums @	19%	25%	56%
- blogs @	5%	14%	81%

^@^ missing data (less than 1%).

**Table 2:** Expression of distress via Internet platforms and attitudes towards gaining support via the Internet in all youths surveyed (N = 1010).

Express distress via online platforms	Express	Do not express	
- social networking platforms@	68%	32%	
- microblogging@	38%	62%	
- forums@	16%	84%	
- blogs@	19%	81%	
**Express distress via any of the above online platforms** @			
Never	24%		
Express via some online platforms	76%		
**Express distress via private means of communication**			
- instant messaging @	65%	35%	
- WhatsApp @	81%	19%	
- phone calls	86%	14%	
- face-to-face	91%	9%	
**Attitudes towards gaining support from the Internet**	**Disagree**	**Neutral**	**Agree**
I have friends with whom I can share my joys and sorrows	13%	24%	62%
I can talk about my problems with my friends @	19%	21%	60%
**Willingness to be approached by online professionals** @	**Unwilling**	**Neutral**	**Willing**
	37%	38%	25%

^@^ missing data (less than 1%).

The characteristics of young people who express distress online constitute helpful information for social work professionals seeking to engage with this population online. Several of the characteristics that distinguished those who express distress online from those who do not are presented in Table 3, which distinguishes the two groups by demographics, and by Internet usage pattern.

**Table 3:** Demographics and Internet usage pattern of youths who do and do not express distress online.

	Do not express online (n = 245)	Express online (n = 756)	Odds ratio^
**Gender**			
Male	63%	41%	0.41 (0.31–0.56)*
Female	37%	59%	1
**Age** @			
17 or younger	28%	26%	1.37 (0.94–2.00)
18–24	38%	51%	1.96 (1.39–2.77)*
25–29	34%	23%	1
**Employment status**			
Student	52%	60%	1
Non-student	48%	40%	0.83 (0.54–1.29)
**Facebook friends**			
Below 150 friends	47%	25%	1
150 to 300 friends	30%	32%	1.95 (1.37–2.78)**
300 or more friends	23%	43%	3.17 (2.19–4.60)**
**Usage of online platforms**			
**- Social networking platforms**			**Adjusted odds ratio**^
Use at least once a day	57%	75%	6.16 (3.64–10.44)**
Use at least once a week	25%	21%	3.96 (2.22–7.04)**
Never use	18%	4%	1
**- Microblogging**		@	
Use at least once a day	18%	39%	3.60 (2.48–5.24)**
Use at least once a week	9%	20%	3.48 (2.15–5.62)**
Never use	73%	40%	1
**- Forums**			
Use at least once a day	15%	20%	2.42 (1.58–3.72)**
Use at least once a week	19%	27%	2.07 (1.41–3.04)**
Never use	66%	53%	1
**- Blogs**			
Use at least once a day	5%	5%	1.16 (0.59–2.25)
Use at least once a week	7%	17%	2.30 (1.36–3.89)**
Never use	87%	79%	1
**Contacting others via private means of communication**			
**- Instant messaging**		@	
Use at least once a day	33%	47%	3.02 (2.10–4.36)**
Use at least once a week	29%	35%	2.48 (1.69–3.63)**
Never use	38%	18%	1
**- WhatsApp**		@	
Use at least once a day	80%	88%	2.79 (1.68–4.63)**
Use at least once a week	7%	6%	2.21 (1.06–4.61)**
Never use	13%	5%	1
**- Phone calls**			
Use at least once a day	71%	70%	1.53 (0.68–3.42)
Use at least once a week	24%	27%	1.66 (0.72–3.83)
Never use	4%	3%	1
**- Face-to-face**			
Use at least once a day	64%	60%	1.49 (0.83–2.66)
Use at least once a week	28%	35%	2.00 (1.08–3.70)**
Never use	8%	5%	1
**Express distress via private means of communication**			
**- Instant messaging**			
Express	27%	78%	9.39 (6.71–13.16)**
Never express	73%	22%	1
**- WhatsApp**			
Express	60%	88%	4.67 (3.30–6.60)**
Never express	40%	12%	1
**- Phone calls**			
Express	76%	90%	2.46 (1.66–3.65)**
Never express	24%	10%	1
**- Face-to-face**			
Express	82%	93%	2.80 (1.78–4.39)**
Never express	18%	7%	1
**Risk behaviors & emotional distress (GHQ)**			
**- Thoughts of suicide**			
Absent	84%	78%	1
Present	16%	22%	1.53 (1.04–2.25)*
**- Help-seeking**			
Have not sought help	21%	18%	1.02 (0.69–1.53)
Have sought informal help	36%	47%	1.61 (1.16–2.25)*
Have sought formal help	4%	3%	1.03 (0.48–2.23)
Not distressed (ref. category)	39%	32%	1

**p < .01.

*p < .05.

^all variables are adjusted for age and gender.

^@^ missing data (less than 1%).

### Use of Internet platforms to voice distress

The results of univariate binary logistic regression analysis showed that the female, student, and 18- to 24-year-old respondents were the most likely to disclose their distress on an online platform, which may have developmental implications. Further, the more Facebook friends a respondent had (OR = 3.17), the likelier he or she was to express distress online, although there were differences between the sexes, with women/girls more likely than men/boys to have large numbers of friends. In this study, 46% of the former and 39% of the latter reported more than 300 Facebook friends.

The results also revealed that the respondents who were more likely to disclose their distress online also reported more frequent contacts with their friends using a variety of means. Those who disclosed distress online were also more likely to use social networking platforms (OR = 6.16) and instant messaging (OR = 3.02) daily, even after adjusting for such confounds as age and gender. They were also more likely to express their distress via instant messaging (OR = 9.39) than via any other platform. Furthermore, this group of youths also engaged in frequent contact with others via WhatsApp (OR = 2.79) or by meeting face-to-face (OR = 2.00) and were likely to be older. Similarly, the youths who expressed distress online, particularly the older ones, were also more likely to express their distress over WhatsApp (OR = 4.67) or in face-to-face conversations (OR = 2.80).

### Voicing distress and profile of risk behaviors

After adjusting for age and gender, the results showed that the group of youths that was more likely to voice distress online was also associated with a higher lifetime prevalence of suicidal ideation (OR = 1.53). With regard to help-seeking behavior, after adjusting for sex, it was found that those who had disclosed distress online were also more likely to seek help from their networks of friends and family (OR = 1.61). Of the respondents who reported expressing distress online, 52% and 41% of the women/girls and men/boys said that they had sought informal help.

An understanding of help-seeking behavior is essential for helping professionals to make good use of resources of the natural support network when planning their outreach strategies. As previously noted, the respondents in this study were separated into four groups on the basis of their help-seeking behavior (see Fig 1), with the non-distressed reference group then compared with each of the three distressed subgroups, namely, the “Did not seek help,” “Seek informal help,” and “Seek formal help” groups.

Compared with their reference group counterparts, member of the informal help group were more likely to be girls/women, students, and aged between 18 and 24. This group of respondents also reported more Facebook friends (300+; OR = 1.43). As shown in Tables 3–5, compared with the reference group, the “Seek informal help” group’s profile was very similar to that of the group of respondents who disclosed distress online, with members of both groups being frequent users of online platforms who frequently express their emotions via online platforms. Relative to the reference group, the “seek informal help” group also had a higher lifetime prevalence of suicidal ideation (OR = 2.09), non-suicidal self-injury (OR = 2.42), high levels of emotional distress (high GHQ scores; OR = 12.78), and being bullied (OR = 2.17).

**Table 4:** Help-seeking behavior among youths with self-perceived distress (compared with the non-distressed group).

	“Did not seek help” group (N = 188)	Adjusted OR^	“Seek informal help” group (N = 446)	Adjusted OR^	“Seek- formal help” group (N = 37)	Adjusted OR^
**Sex**						
Male	57%	1	39%	1	38%	1
Female	43%	0.79 (0.55–1.13)	61%	1.63 (1.22–2.17)**	62%	1.71 (0.85–3.44)
**Age**	@		@			
Below 17	18%	1	28%	1	22%	1
18–24	53%	2.35 (1.47–3.75)***	52%	1.51 (1.07–2.11)*	51%	1.90 (0.80–4.51)
25–29	28%	1.50 (0.90–2.50)	20%	0.70 (0.48–1.03)	27%	1.20 (0.46–3.17)
**Employment status**						
Student	54.8%	1	64.1%	1	48.6%	1
Non-student	45.2%	0.76 (0.43–1.35)	35.9%	0.64 (0.40–1.00)*	51.4%	0.96 (0.34–2.66)
**Facebook friends**						
Below 150 friends	31%	1	26%	1	41%	1
150 to 300 friends	34%	1.32 (0.84–2.06)	32%	1.37 (0.95–1.98)	32%	0.88 (0.39–1.98)
300 friends or more	35%	1.15 (0.74–1.78)	42%	1.43 (1.01–2.03)*	27%	0.59 (0.25–1.38)
**Usage of online platforms**						
**- Social networking**						
Use at least once a day	71%	1.08 (0.56–2.06)	73%	1.91 (1.05–3.48)*	65%	0.55 (0.19–1.56)
Use at least once a week	20%	0.90 (0.43–1.85)	22%	1.77 (0.93–3.38)	22%	0.57 (0.17–1.90)
Never use	9%	1	4%	1	14%	1
**- Microblogging (e.g., Twitter, Weibo, etc.)**					@	
Use at least once a day	32%	1.08(0.72–1.63)	38%	1.22 (0.88–1.69)	28%	0.82 (0.36–1.87)
Use at least once a week	16%	0.88 (0.53–1.45)	17%	0.88 (0.59–1.31)	22%	1.08 (0.44–2.64)
Never use	52%	1	46%	1	50%	1
**- Forums**	@				@	
Use at least once a day	21%	1.15 (0.71–1.87)	19%	1.45 (0.97–2.15)	17%	1.15 (0.43–3.10)
Use at least once a week	23%	1.14 (0.73–1.79)	28%	1.65 (1.16–2.34)**	28%	1.48 (0.66–3.35)
Never use	56%	1	53%	1	56%	1
**- Blogs**					@	
Use at least once a day	6%	1.23 (0.57–2.64)	4%	0.86 (0.43–1.72)	8%	2.22 (0.60–8.18)
Use at least once a week	11%	0.88 (0.50–1.56)	16%	1.29 (0.85–1.95)	25%	2.32 (1.01–5.36)**
Never use	83%	1	80%	1	0%	1
**Express distress via online platforms**						
**- Social network platforms**						
Express	64%	0.99 (0.68–1.45)	73%	1.25 (0.91–1.71)	62%	0.77 (0.38–1.57)
Do not express	36%	1	27%	1	38%	1
**- Micro-blogging**	@		@		@	
Express	33%	1.07 (0.73–1.58)	44%	1.45 (1.07–1.96)*	37%	1.10 (0.52–2.29)
Do not express	67%	1	56%	1	63%	1
**- Forums**	@		@			
Express	13%	0.74 (0.44–1.24)	18%	1.21 (0.82–1.78)	16%	1.04 (0.41–2.63)
Do not express	87%	1	82%	1	84%	1
**-Blogs**	@		@		@	
Express	17%	1.18 (0.72–1.91)	21%	1.38 (0.95–2.02)	33%	2.51 (1.17–5.36)*
Do not express	83%	1	79%	1	67%	1
**Proportion using online platform to express distress**						
	@		@		@	
Do not express on any online platform	28%	1	20%	1	28%	1
Some sort of online platform expression	72%	0.91 (0.60–1.36)	80%	0.69 (0.49–0.97)*	72%	1.10 (0.50–2.40)
**Express distress via private means of communication**						
**- Instant messaging**			@			
Express	62%	0.96 (0.66–1.40)	69%	1.17 (0.87–1.59)	57%	0.71 (0.36–1.42)
Do not express	38%	1	31%	1	43%	1
**-WhatsApp**			@			
Express	78%	1.11 (0.72–1.71)	92%	1.81 (1.25–2.63)**	78%	1.01 (0.44–2.32)
Do not express	22%	1	8%	1	22%	1
**- Phone calls**						
Express	77%	0.70 (0.44–1.11)	8%	2.20 (1.40–3.47)**	89%	1.41 (0.47–4.22)
Do not express	23%	1	92%	1	11%	1
**-Face-to-face**						
Express	86%	0.82 (0.48–1.42)	94%	1.83 (1.10–3.07)*	95%	2.01 (0.46–8.74)
Do not express	14%	1	6%	1	5%	1
**Willingness to be approached by online professionals**						
Neutral	37%	1	38%	1	30%	1
Unwilling	36%	1.04 (0.69–1.58)	38%	1.09 (0.78–1.50)	24%	0.88 (0.35–2.21)
Willing	27%	1.23 (0.78–1.95)	24%	1.11 (0.77–1.61)	46%	2.67 (1.19–6.01)*

*** p < .001.

**p < .01.

*p < .05.

^all variables are adjusted for age and sex.

^@^ missing data (less than 1%).

**Table 5:** Risk behaviors and emotional distress among youths with self-perceived distress (compared with non-distressed group).

	“Did not seek help” group (N = 188)	Adjusted OR^	“Seek informal help” group (N = 446)	Adjusted OR^	“Seek formal help” group (N = 37)	Adjusted OR^
**Suicidal thoughts**						
Absent	74%	1	77%	1	70%	1
Present	26%	2.36 (1.49–3.72)***	23%	2.09 (1.42–3.09)***	30%	2.87 (1.32–6.24)**
**Intentional self-injury**						
Absent	86%	1	88%	1	81%	1
Present	14%	2.98 (1.59–5.57)**	12%	2.42 (1.38–4.23) **	19%	4.27 (1.65–11.08)**
**More than 5 alcoholic drinks per occasion**						
Absent	70%	1	72%	1	62%	1
Present	30%	1.13 (0.74–1.72)	28%	1.33 (0.94–1.87)	38%	1.87 (0.88–4.00)
**Smoke more than 5 cigarettes per day**						
Absent	87%	1	93%	1	84%	1
Present	13%	0.99 (0.57–1.73)	7%	0.66 (0.40–1.09)	16%	1.52 (0.57–4.03)
**Drug/Substance abuse**						
Absent	96%	1	99%	1	95%	1
Present	4%	0.92 (0.36–2.39)	1%	0.22 (0.06–0.80)*	5%	1.62 (0.34–7.76)
**Gambling**						
Absent	70%	1	75%	1	70%	1
Present	30%	0.89 (0.58–1.37)	25%	0.93 (0.65–1.31)	30%	1.00 (0.44–2.25)
**Debt problems**						
Absent	88%	1	95%	1	95%	1
Present	12%	2.87 (1.42–5.78)**	5%	1.47 (0.74–2.93)	5%	1.26 (0.27–5.94)
**Unsafe sex**			@			
Absent	86%	1	93%	1	81%	1
Present	14%	2.49 (1.32–4.71)**	7%	1.59 (0.87–2.90)	19%	4.47 (1.64–12.18)**
**STD**						
Absent	100%	-	100%	1	97%	1
Present	0%	-	0%	1.25 (0.07–21.25)	3%	13.09 (0.75–229.12)
**Unintended pregnancy**						
Absent	98%	1	99%	1	100%	-
Present	2%	4.62 (0.81–26.26)	1%	2.33 (0.46–11.86)	0%	-
**Abortion**						
Absent	99%	1	99%	1	100%	-
Present	1%	2.02 (0.28–14.56)	1%	1.03 (0.17–6.25)	0%	-
**Bullied others**						
Absent	87%	1	89%	1	89%	1
Present	13%	2.48 (1.31–4.67)**	11%	2.38 (1.36–4.19)**	11%	2.40 (0.75–7.65)
**Bullied by others**						
Absent	81%	1	84%	1	68%	1
Present	19%	2.53 (1.48–4.32)**	16%	2.17 (1.36–3.46)**	32%	5.69 (2.57–12.59)***
**Criminal offenses**						
Absent	95%	1	99%	1	89%	1
Present	5%	2.09 (0.78–5.64)	1%	0.79 (0.26–2.34)	11%	6.05 (1.63–22.47)**
**Emotional distress**						
Low GHQ	94%	1	93%	1	89%	1
High GHQ	6%	10.7 (2.34–48.83)**	7%	12.78 (3.03–53.94)**	11%	19.96 (3.51–113.41)**

***p < .001.

**p < .01.

*p < .05.

^all variables are adjusted for age and gender.

^@^ missing data (less than 1%).

Interestingly, the “Did not seek help” and “Seek formal help” groups had a similar risk profile relative to the reference group. Both groups had a higher lifetime prevalence of suicidal ideation, non-suicidal self-injury, unsafe sex, high levels of emotional distress (high GHQ scores), and being bullied (please refer to Table 4 for the adjusted ORs). However, members of the “Did not seek help” group were more likely to have debt (OR = 2.87) and bullying problems (OR = 2.48) than members of the reference group, whereas members of the “Seek formal help” group were more likely to have committed a criminal offense (OR = 6.05). The latter were also more willing to be approached by online professionals (OR = 2.67), possibly because they were in greater need of professional support than members of the other groups.

## Discussion

To the best of our knowledge, this is the first study to investigate help-seeking behavior through possible means of Internet engagement and to examine the online expression of distress by young people using a representative sample of Chinese youths. Its results indicate that young female students aged 18 to 24 with strong online and personal networks are the most likely to express emotional distress online, which is helpful information for helping professionals seeking to identify the population of youths most likely to view the Internet as an emotional outlet. With the strong personal and well-developed online networks, these professionals may be able to increase their influence by attempting to connect with youths and their friends online and/or promoting their services online or making them accessible via the Internet. For instance, helping professionals could recruit volunteers to serve as Internet ambassadors to safeguard the online space to promote a more caring Internet culture. These volunteers could visit online forums or Facebook groups to leave caring messages and offer words of kindness to those who appear to be in distress.

The study’s results also suggest that professionals should view those who have either never sought help or sought informal help alone as targets for engagement. In this research, respondents who were distressed had a higher prevalence of several risk behaviors compared with their non-distressed counterparts. More importantly, approximately 20% of the distressed youths surveyed (i.e., members of the “Did not seek help” group) had sought neither formal nor informal help despite expressing their distress online. According to the GPM, helping professionals can act as gatekeepers to identify youths who make negative expressions online and offer them formal services. The distressed youths in the current study were divided into groups on the basis of their help-seeking behavior (see Fig 1). Relative to the “Did not seek help” group, the “seek informal help” group exhibited a stronger tendency to express distress via online platforms. However, the former agreed that online platforms are a good venue for retaining anonymity. These findings shed light on what professionals can do to earn trust and build rapport during the engagement process. The adoption of the Internet as an outreach platform opens up a new range of possibilities and areas for discussion for the providers of youth services.

This study has several limitations that must be acknowledged. First, as it involved a telephone-based survey based on randomly generated mobile phone numbers, youths who do not use a mobile phone were excluded. Second, the response rate was less than ideal, possibly because of the relatively extensive length of the survey questionnaire. Third, the survey comprised only self-report items, which are known to be prone to recall bias, and there is thus no way to verify how accurately the findings reflect the true Internet behavior of Hong Kong youths. Finally, one important issue has been left unaddressed by our study, that is, the question of whether young people are likely to search online for health or risk behavior information prior to their disclosure of distress online. If so, knowledge of what kinds of websites they browse (e.g., sites discussing suicide methods) would be helpful for professionals seeking to better target their outreach efforts [25]. Further research in this area is necessary.

**Fig 2:**
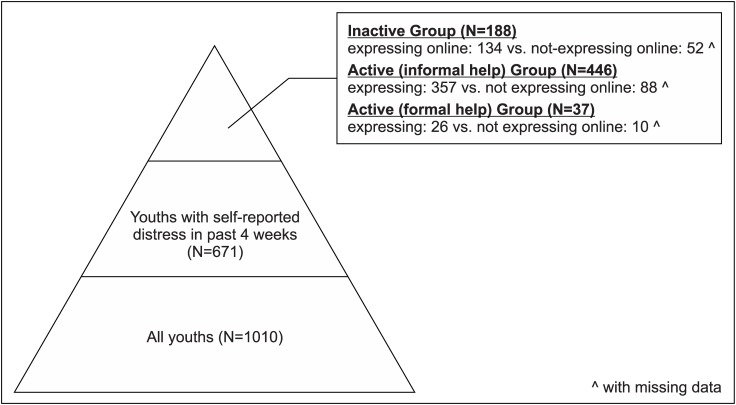
Pyramid model of the survey.

To the best of our knowledge, there has been only few evaluative studies to date concerning an online outreach intervention restricted to a specific population (i.e., Chinese men who have sex with men) [18], which implies that online outreach has the potential to be expanded to other problem areas (e.g., cyberbullying and youth social withdrawal) [26–27]. Researchers should consider cooperating with social media companies (e.g., Facebook, Google, Baidu) to make relevant information accessible to those who need it. Social media platforms play a supplementary role to existing youth services, providing a substitute means of engagement. Youth service providers should take note of the nature of online engagement and consider providing timelier, more accessible services to youths at risk of mental health problems. Advanced technologies can also be utilized to improve the online detection of at-risk youths [28–30]. All of these strategies should be incorporated into future deliberations on youth service provision in the Internet era. If their features are used properly, social media can be an effective means to connect with the previously disconnected.

(Please refer to Fig1.tif for Fig 1. Categorization of the four groups for DV2 and Fig2.tif for Fig 2. Pyramid model of the survey.)
